# Chest CT features of coronavirus disease-19 (COVID-19) pneumonia: which findings on initial CT can predict an adverse short-term outcome?

**DOI:** 10.1259/bjro.20200016

**Published:** 2020-06-19

**Authors:** Arshed Hussain Parry, Abdul Haseeb Wani, Naveed Nazir Shah, Mudasira Yaseen, Majid Jehangir

**Affiliations:** 1 Department of Radiodiagnosis, Sher-i-Kashmir Institute of Medical Sciences, Srinagar, Jammu and Kashmir, India; 2 Department of Radiodiagnosis, Government Medical College, Srinagar, Jammu and Kashmir, India.; 3 Department of Chest Medicine, Government Medical College, Srinagar, Jammu and Kashmir, India; 4 Department of Anesthesiology and Critical Care Medicine, Sher-i-Kashmir Institute of Medical Sciences, Srinagar, Jammu and Kashmir, India.; 5 Department of Radiodiagnosis, Government Medical College, Srinagar, Jammu and Kashmir, India.

## Abstract

**Objective:**

To study the spectrum of chest CT features in coronavirus disease-19 (COVID-19) pneumonia and to identify the initial CT findings that may have the potential to predict a poor short-term outcome.

**Methods:**

This was a retrospective study comprising 211 reverse transcriptase-polymerase chain reaction (RT-PCR) positive patients who had undergone non-contrast chest CT. Prevalence, extent, pattern, distribution and type of abnormal lung findings were recorded. Patients with positive CT findings were divided into two groups; clinically stable (requiring in-ward hospitalization) and clinically unstable [requiring intensive care unit (ICU) admission or demised] based on short-term follow-up.

**Results:**

Lung parenchymal abnormalities were present in 42.2% (89/211) whereas 57.8% (122/211) cases had a normal chest CT. The mean age of clinically unstable patients (63.6 ± 8.3 years) was significantly different from the clinically stable group (44.6 ± 13.2 years) (*p*-value < 0.05). Bilaterality, combined involvement of central–peripheral and anteroposterior lung along with a higher percentage of the total lung involvement, presence of crazy paving, coalescent consolidations with air bronchogram and segmental pulmonary vessel enlargement were found in a significantly higher proportion of clinically unstable group (ICU/demised) compared to the stable group (in-ward hospitalization) with all *p* values < 0.05.

**Conclusion:**

Certain imaging findings on initial CT have the potential to predict short-term outcome in COVID-19 pneumonia. Extensive pulmonary abnormalities, evaluated by combined anteroposterior, central–peripheral and a higher percentage of the total lung involvement, indicate a poor short-term outcome. Similarly, the presence of crazy paving pattern, consolidation with air bronchogram and segmental vascular changes are also indicators of poor short-term outcome.

**Advances in knowledge:**

Certain findings on initial CT can predict an adverse short-term prognosis in COVID-19 pneumonia.

## Introduction

Coronavirus disease 2019 (COVID-19), which emerged in Wuhan, China towards the end of 2019 and spread unabated across the globe, posing daunting challenges to the healthcare system, compelling the World Health Organization (WHO) to declare this outbreak as a public health emergency of international concern on 30 January, 2020. The total number of cases of COVID-19 has surpassed 4.6 million globally with 311,847 deaths. The United States of America (USA) has reported the highest number of cases and deaths among all the nations as of May 19, 2020. India has reported 96,169 cases with 3029 deaths as of May 19, 2020 with an estimated mortality of 3.14% against the global reported mortality rate of 6.75%.^1^

COVID-19 is a highly contagious viral disease caused by severe acute respiratory syndrome coronavirus 2 (SARS-CoV-2) which is a member of betacorona viruses which are enveloped single-stranded RNA viruses.^2^ The disease spreads via the respiratory route. Clinical presentation varies from asymptomatic, through symptomatic cases to critically ill. The commonest symptoms include fever, cough, sore throat and dyspnea.^3^ The reference standard for the diagnosis of SARS-CoV-2 infection is real-time reverse transcription polymerase chain reaction (RT-PCR) performed on respiratory tract specimens. However, owing to various limitations like sample collection, transportation, type of sample tested, processing time and performance of diagnostic kit, the sensitivity of RT-PCR varies from 60 to 71%.^4,5^ Though CT is not a screening tool for detection of COVID-19, however, the role of CT in the diagnosis, triage, and prognostication of patients with COVID-19 infection continues to be refined. Imaging may serve as a complementary tool to detect early pathological lung changes in RT-PCR negative patients which can ensure timely detection and immediate care to contain the transmission of this disease in the population. Additionally, it is a feasible modality to assess the severity and course of the disease. Due to its low sensitivity, the chest X-ray is not a suitable tool to detect COVID-19 lung pathology. CT is an excellent modality to conclusively confirm or refute lung pathology in suspected patients. CT features of COVID-19 closely resemble other viral pneumonias and include ground glass opacities, crazy paving pattern, consolidations with peripheral and basal predominance which are already documented in literature.^6–8^

It has been suggested that certain imaging features may be more prevalent in severely ill and expired COVID-19 patients compared to the patients undergoing routine in-ward hospitalization.^9^ This endeavor aimed to study the CT features of COVID-19 pneumonia in Indian patients and to assess the findings on initial CT that could potentially predict an adverse short-term outcome.

## Methods and materials

### Patient cohort and study design

This was a retrospective study wherein we reviewed chest CT scans of 211 RT-PCR positive patients from 16 March to 25 April, 2020 in our hospital which was designated as COVID-19 Care Centre (CCC) with separate inpatient, intensive care unit (ICU) and quarantine facilities. The study was approved by the Institutional Review Board. Requirement of informed patient consent was waived by the Institutional Ethical Committee.

Medical records and chest CT scans of 211 patients who had been referred to our CCC from various districts were analyzed. The diagnosis of COVID-19 infection was confirmed by RT-PCR performed on nasopharyngeal or oropharyngeal swab. In some critically ill patients, RT-PCR was performed on endotracheal aspirate.

Patient demographics and specific clinical information were obtained from clinical records. Patients with chest CT findings at admission were further divided into two groups based on disease severity. This decision was based on the WHO interim guidance for clinical management of patients with COVID-19.^10^ Group 1 (clinically stable patients) with mild disease, which was defined as (a) respiratory rate <30 breaths /min (b) oxygen saturation (SpO2) >90% and (c) absence of signs of respiratory failure, acute respiratory distress syndrome (ARDS), or shock. This group was admitted in a routine ward. Group 2 (clinically unstable patients) with severe form of disease, which was defined as (a) respiratory rate ≥30 breaths/min, or (b) oxygen saturation (SpO2) ≤90%, or (c) respiratory failure needing mechanical ventilation, or (d) ARDS, or (e)shock.^10^ This group of patients required ICU admission or subsequently expired. The patients were followed for a short period of time (average 17 days; range 12–22 days). Comparison of demographic, clinical and various CT imaging features were made between the two distinct clinical groups.

### CT acquisition protocol and image interpretation

Patients were subjected to CT chest after collection of a nasopharyngeal or oropharyngeal swab. Chest CT was performed on an average 4.3 days (range 1–7 days) after symptom onset. All the CT scans were performed on 16-Slice Siemens Somatom, Erlangen, Germany, Emotion Multidetector CT with a single breath-hold using 16 × 0.6 collimation, 100–120 kVp, and 90–130 mAs using automated exposure control with a beam pitch of 1.5. The image was acquired in 5 mm thickness and reconstructed using reconstruction increment of 0.7 mm into 1 mm thick slices using a sharp kernel (B70s) algorithm. The images were viewed in lung window settings at a window width of 1200–1500 HU and centering of −500 to −600 HU and mediastinal window using a window width of 300-400H U and centering of 40 HU. Decontamination of the CT suite consisted of surface disinfection with 70% ethanol or 0.1% sodium hypochlorite. After each CT examination, passive air exchange was performed for 60 min.

Two radiologists with 8 and 7 years of experience in reading chest CT images evaluated CT images on an Apple Mac workstation in a satellite room. Imaging was reviewed independently and final decisions reached by consensus. In case of any disagreements between the two primary interpreting radiologists, a third experienced radiologist with 21 years experience adjudicated the final decision. The readers assessed the presence, location, extent and density of lung parenchymal abnormality. The location of lesions was specified with regards to involvement of one lung or bilateral involvement. Lobar distribution of parenchymal abnormalities was also assessed. The distribution was also dichotomized with regards to the central and peripheral lung. The central lung was defined as the inner two-third of the lung tissue, and peripheral lung was defined as outer one-third of the lung. The distribution of lung abnormalities was also noted with regards to the anterior and posterior location (lung tissue anterior to a line drawn midway on axial CT was defined as anterior and the portion behind it was defined as posterior). Lung lesions were categorized using Fleischner society glossary of terms for thoracic imaging.^11^ Ground glass opacity (GGO) was defined as an increase in density of lung with visualization of bronchial and vascular structures through it, whereas consolidation was defined as increased density of lung tissue through which vascular and bronchial structures were not visible. The readers also evaluated the percentage of total lung involvement by dividing the lungs into three zones (upper, middle, lower). The upper zone was defined as the portion of lungs above the level of the carina; the middle zone between the carina and the inferior pulmonary vein and the lower zone below the level of inferior pulmonary vein.^12^ Visual assessment of the percentage of involvement of each lung zone was assessed followed by summation of all six zones to get the percentage of the lung involvement. Furthermore, the readers also evaluated presence of associated airway, vascular, pleural, pericardial and mediastinal abnormalities. A vascular enlargement was considered when the segmental or subsegmental vessel was enlarged in comparison to the contralateral side or when the vessel diameter exceeded 3 mm. We assessed the initial CT imaging features between the two distinct groups of clinically stable (in-ward) patients and the clinically unstable (ICU/demised) patients.

### Statistical analysis

Statistical analysis was performed using the Statistical Package for the Social Sciences (SPSSInc. Chicago, IL, v. 21.0) and Open source epidemiologic statistics for public health (EPI; Dean AG, Sullivan KM, Soe MM, MIT). Continuous variables were expressed as means and standard deviations, whilst categorical variables were expressed as counts and percentages. Statistical calculations were performed using Fisher’s exact test for categorical variables and the two sample Student’s *t* test for continuous variables. A *p*-value less than 0.05 was considered statistically significant. The agreement between two interpreting radiologists for CT findings was evaluated with the κ method (according to Landis and Koch 0: poor agreement; 0.01–0.20: slight agreement; 0.21–0.40: fair agreement; 0.41–0.60: moderate agreement; 0.61–0.80: substantial agreement; 0.81–1.0: almost perfect agreement).

## Results

### Clinical characteristics and demographics

**Figure 1.**
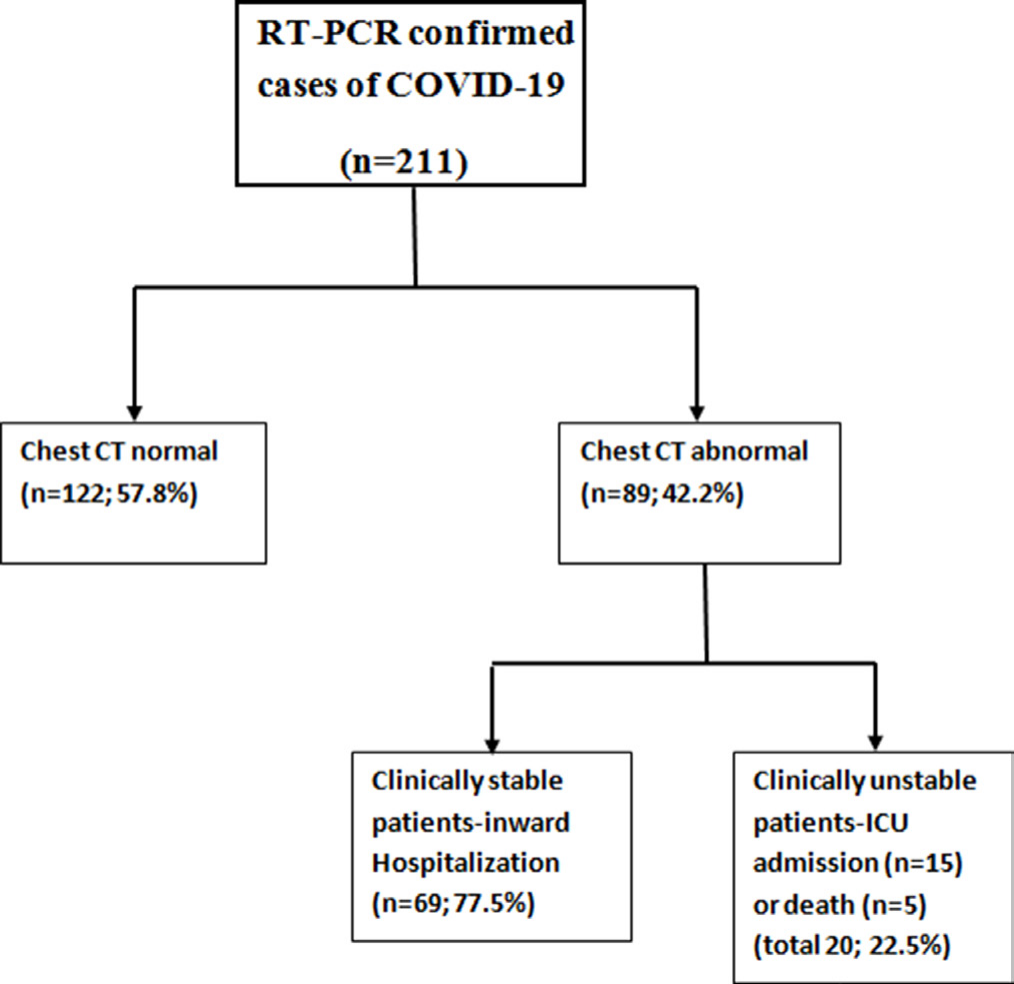
Flow chart of COVID-19 patients who underwent chest CT. COVID-19, Corona virus disease 2019; ICU, intensive care unit; RT-PCR, reverse transcription-polymerase chain reaction.

Among the total study cohort of 211 patients, 70.6% (149/211) were males and 29.4% (62/211) were females with a mean age of 47.7 ± 13.4 years. 57.8% (122/211) patients had a normal chest CT. The remaining (89/211; 42.2%) had abnormal chest CT and were divided into two groups based on short-term clinical follow up (average follow-up period 17 days ranging from 12 to 22 days) (Figure 1). 77.5% (69/89) were clinically stable and were admitted in the ward. 22.5% (20/89) were clinically unstable and were admitted to ICU and got intubated. Among them, five died; two of them had diabetes and one had chronic liver disease. Age of the clinically stable group (44.6 ± 13.2) was significantly lower than the unstable group (63.6 ± 8.3) (*p* < 0.05). There was no significant statistical difference in gender between the two groups (*p*-value > 0.05) (Table 1). The most common symptoms on admission were fever, cough, fatigue, myalgia and expectoration (Table 2). Gastrointestinal and neurological symptoms were less common. The time from illness onset to hospital admission (diagnosis) was 4.3 (1–7) days.

**Table 1.** Comparison of demographic and CT findings in two clinical groups of COVID-19 pneumonia

	**Parameter**	**Overall (*n* = 89)**	**Clinically stable group (No ICU admission or death) (*n* = 69; 77.5%)**	**Clinically unstable group (ICU admission or death) (*n* = 20; 22.5%)**	*p*-value
**DEMOGRAPHICS**	**Age (Mean ± SD)**	49.1 ± 14.1	44.6 ± 13.2	63.6 ± 8.3	**0.026^+^**
	**Sex**	*M* = 62 *F* = 27	*M* = 47 *F* = 22	*M* = 15 *F* = 5	>0.05
**EXTENT/BURDENOFDISEASE**	**Laterality Bilateral Unilateral**	68 (76.4%) 21 (23.6%)	49 (71%) 20 (29%)	19 (95%) 1 (5%)	**0.034**
	**Lobar involvement**	RUL 73 (82%) RML 51 (57.3%) RLL 68(76.4%) LUL 65 (73%) LLL 80(89.8%)	RUL 55 (79.7%) RML 38 (55.1%) RLL 53 (76.8%) LUL 51 (73.9%) LLL 61 (88.4%)	RUL 16 (80%) RML 12 (60%) RLL 17 (85%) LUL 13 (65%) LLL 19 (95%)	>0.05
	**Axial distribution**	Peripheral 65 (73%) Peripheral + Central 24 (27%)	Peripheral 62 (89.9%) Peripheral + Central 7 (11.1%)	Peripheral 3 (15%) Peripheral + Central 17 (85%)	**0.00001**
	**AP distribution**	Posterior 52 (58.4%) Anteroposterior 37 (41.6%)	Posterior 46 (66.7%) Anteroposterior 23 (33.3%)	Posterior 6 (30%) Anteroposterior 14 (70%)	**0.0046**
	**Percentage of total lung involvement**	21.3 ± 8.4	17.1 ± 7.3	39.1 ± 13.2	**0.0034^+^**
**PULMONARY PARENCHYMAL DAMAGE**	**Pure GGO**	89 (100%)	69 (100%)	20 (100%)	>0.05
	**Crazy paving pattern**	29 (32.6%)	15 (21.7%)	14 (70%)	**0.0001**
	**Consolidation**	42 (47.2%)	26 (37.7%)	16 (80%)	**0.001**
	**Air bronchogram sign**	22 (24.7%)	9 (13%)	13 (65%)	**<0.05**
	**Vessel enlargement**	60 (67.4%)	42 (60.9%)	18 (90%)	**0.026**
	**Subpleural curvilinear lines**	16 (18%)	12 (17.4%)	4 (20%)	>0.05
	**Reticulations**	26 (29.2%)	21 (30.4%)	5 (25%)	>0.05
	**Reverse halo sign**	16 (18%)	15 (21%)	1 (5%)	>0.05

AP, anteroposterior; COVID-19, Corona virus disease 2019; GGO, ground glass opacity; ICU, intensive care unit; SD, standard deviation.

**Table 2.** Clinical characteristics of COVID-19 infected patients at admission

**Clinical characteristic**	**Negative CT at the time of admission (*n* = 122**)	**Abnormal CT at the time of admission**	
		**In-ward/clinically stable patients (*n* = 69)**	**ICU/clinically unstable patients (*n* = 20)**
**Asymptomatic**	**77 (63.1%)**	**8 (11.6%)**	**/**
**Fever (>37.3^0^C)**	**32 (26.2%)**	**59 (85.5%)**	**18 (90%)**
**Cough**	**11 (9%)**	**42 (60.9%)**	**11 (55%)**
**Expectoration**	**3 (2.4%0**	**9 (13%)**	**4 (20%)**
**Shortness of breath**	**/**	**3 (4.3%)**	**14 (70%)**
**Fatigue**	**12 (9.8%)**	**21 (30.4%)**	**7 (35%)**
**Myalgia**	**6 (4.9%)**	**11 (15.9%)**	**3 (15%)**
**Nausea/vomiting**	**3 (2.4%)**	**4 (5.8%)**	**1 (5%)**
**Diarrhea**	**7 (5.7%)**	**4 (5.8%)**	**1 (5%)**
**Abdominal pain**	**3 (2.5%)**	**2 (2.9%)**	**1 (5%)**
**Headache**	**6 (4.9%)**	**5 (7.2%)**	**2 (10%)**
**Dysguesia**	**2 (1.6%)**	**1 (1.4%)**	**/**
**Hyposmia/anosmia**	**/**	**1 (1.4%)**	**/**
**Dizziness**	**/**	**3 (4.3%)**	**2 (10%)**

COVID-19, Corona virus disease 2019; ICU, intensive care unit.

**Figure 2.**
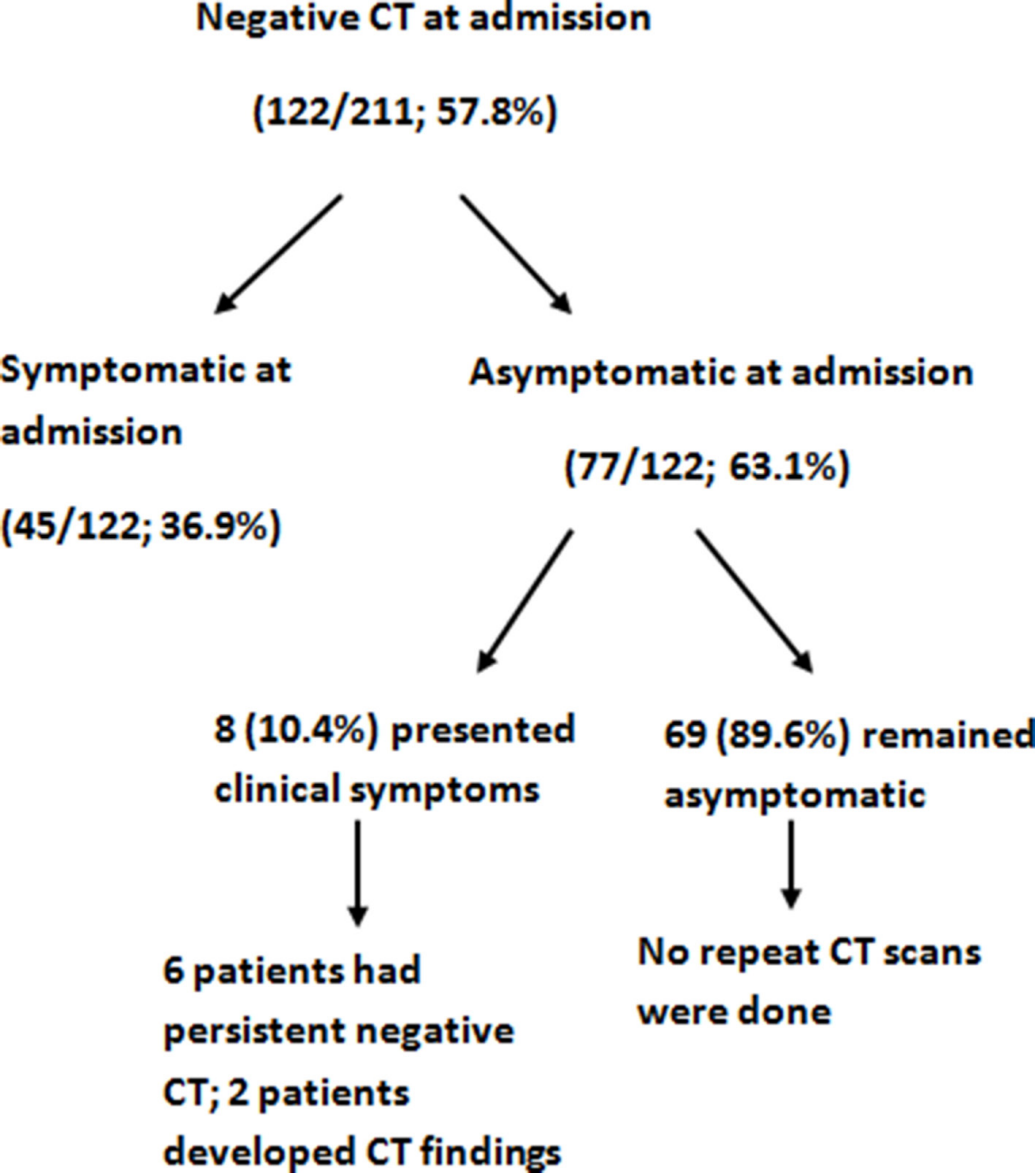
Flow chart of patients with negative/normal chest CT at admission.

However, the majority (77/122; 63.1%) of patients with initial negative CT were asymptomatic at admission, among which 8 (10.4%) patients presented with symptoms 7.2 ± 3.8 days after admission (Figure 2).

### Chest CT findings

**Figure 3.**
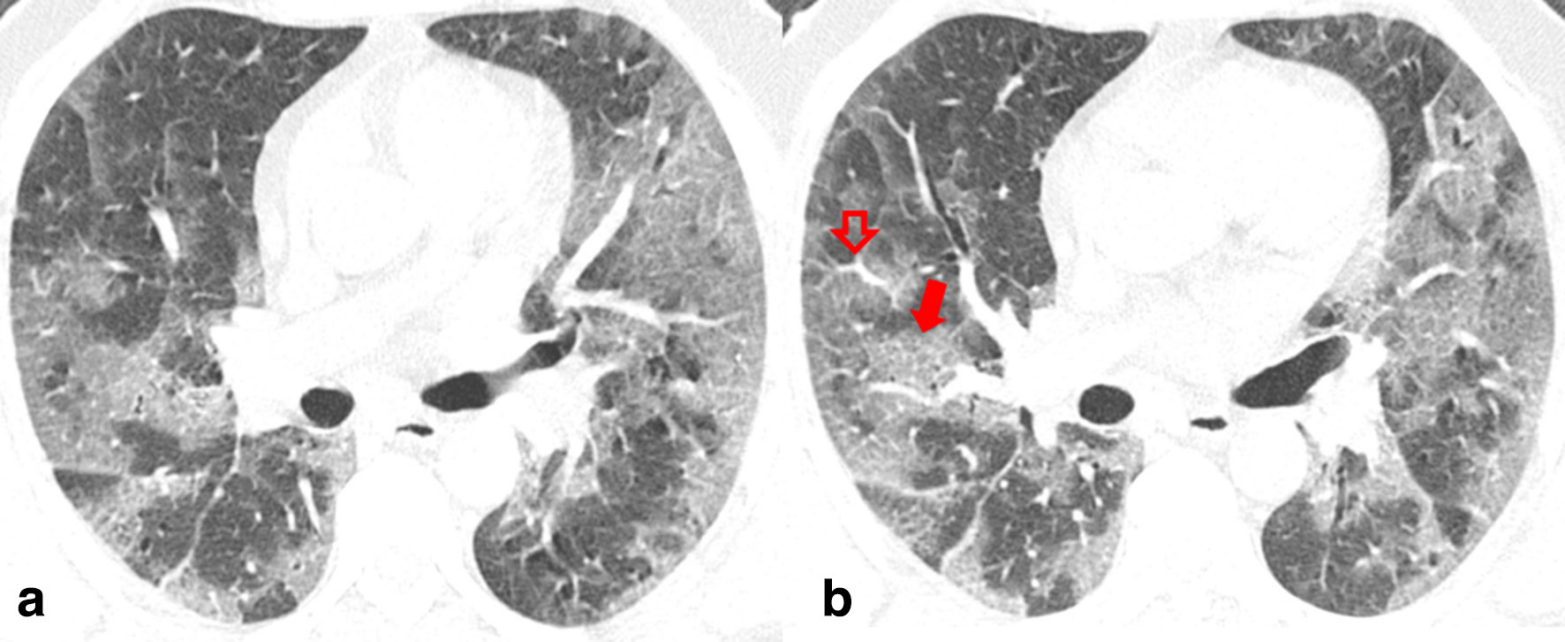
Two consecutive non-contrast axial chest CT images in lung window settings of a 62-year-old male COVID-19 positive patient showing multiple patchy peripherally and centrally distributed ground glass opacities with crazy paving pattern denoted by the solid arrow. Segmental vessel dilatation is also seen, denoted by a hollow arrow. The patient required mechanical ventilation (clinically unstable) and subsequently expired. COVID-19, Corona virus disease 2019

There was almost perfect agreement (Cohen’s κ of 0.84) in reading CT images between the two radiologists. Lung parenchymal abnormalities on chest CT imaging were observed in 89 patients. Bilateral lung involvement was significantly more common in the unstable group (95%; 19/20) than stable group (71%; 49/69). In terms of axial distribution, peripheral lung involvement was seen in both the stable and unstable group of patients with significantly higher involvement of central lung in the clinically unstable group (85%; 17/20) (*p* < 0.05) (Figure 3). Similarly, with regards to AP distribution, combined involvement of posterior and anterior lung was seen in a significantly higher number of patients in unstable group (*p*-value < 0.05). A significantly higher percentage of total lung involvement was noted in the clinically unstable group (39.1 ± 13.2) than the stable group of in-ward patients (17.1 ± 7.3) (*p*-value = 0.0034).

**Figure 4.**
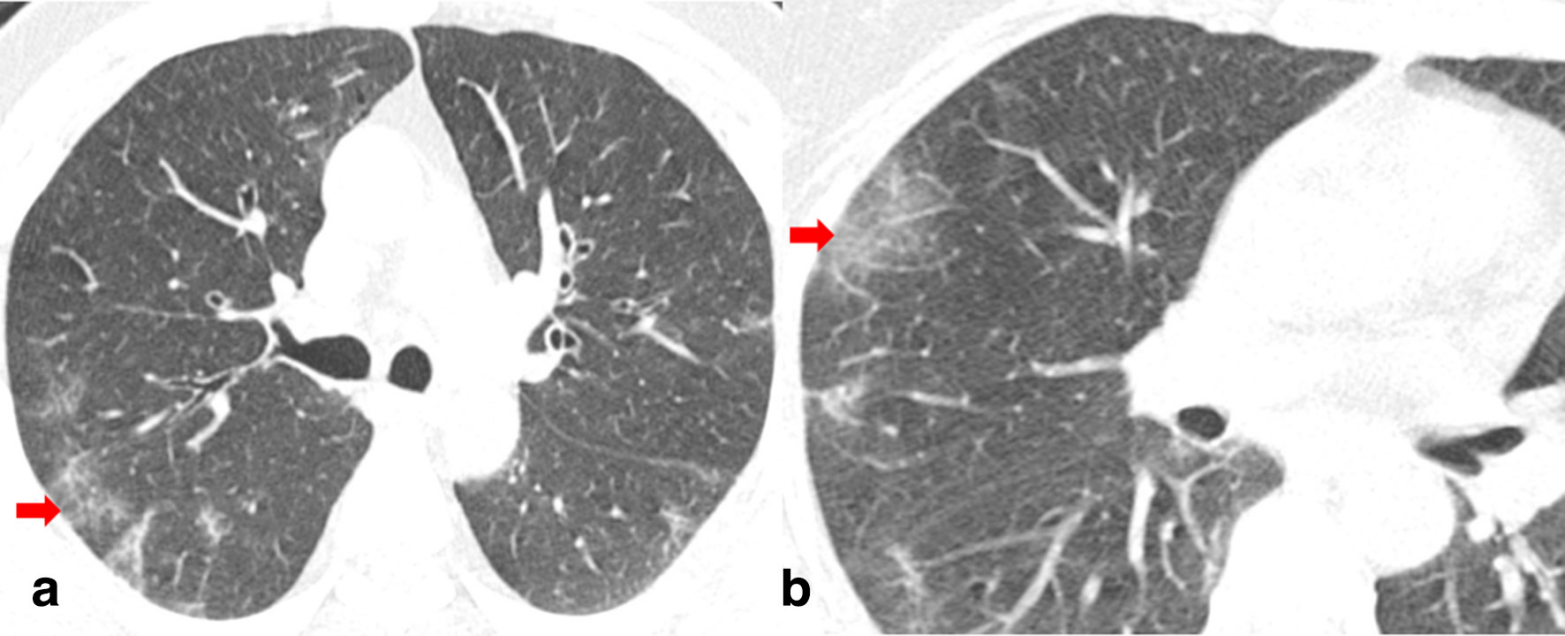
Non-contrast chest CT axial image (a) in the lung window setting of a 49-year-old male COVID-19 positive patient admitted in routine ward (clinically stable), showing few bilateral patchy ground glass opacities with fine reticulations (red arrow). Fig. b is the zoomed-in view of the same patient at a lower level showing GGO (red arrow) with fine reticulations. COVID-19, Corona virus disease 2019; GGO, ground glass opacity.

**Figure 5.**
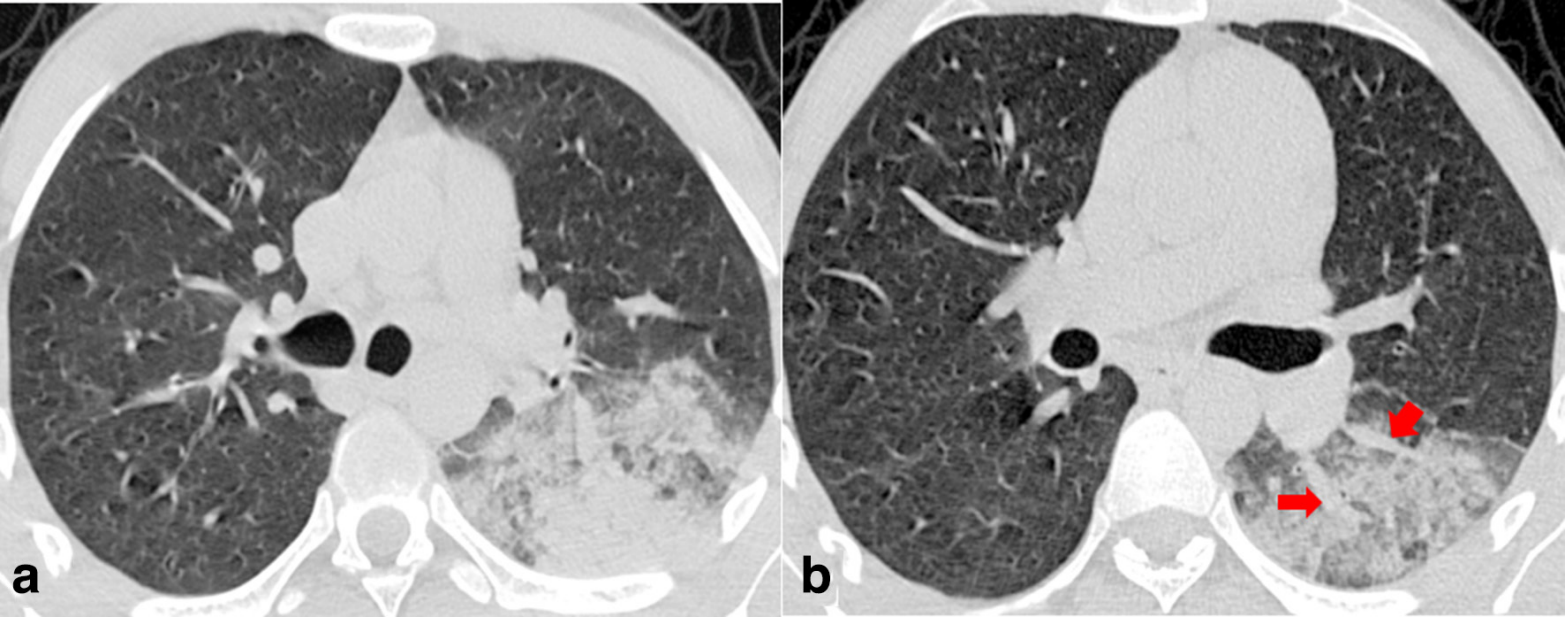
Non-contrast chest CT axial image (a) in a 24-year-old COVID-19 positive male patient showing coalescent consolidative opacity in the superior segment of the left lower lobe. Vessel dilatation sign is seen in (b) denoted by arrow. The patient was clinically unstable and required ventilation in the ICU. COVID-19, Corona virus disease 2019; ICU, intensive care unit.

**Figure 6.**
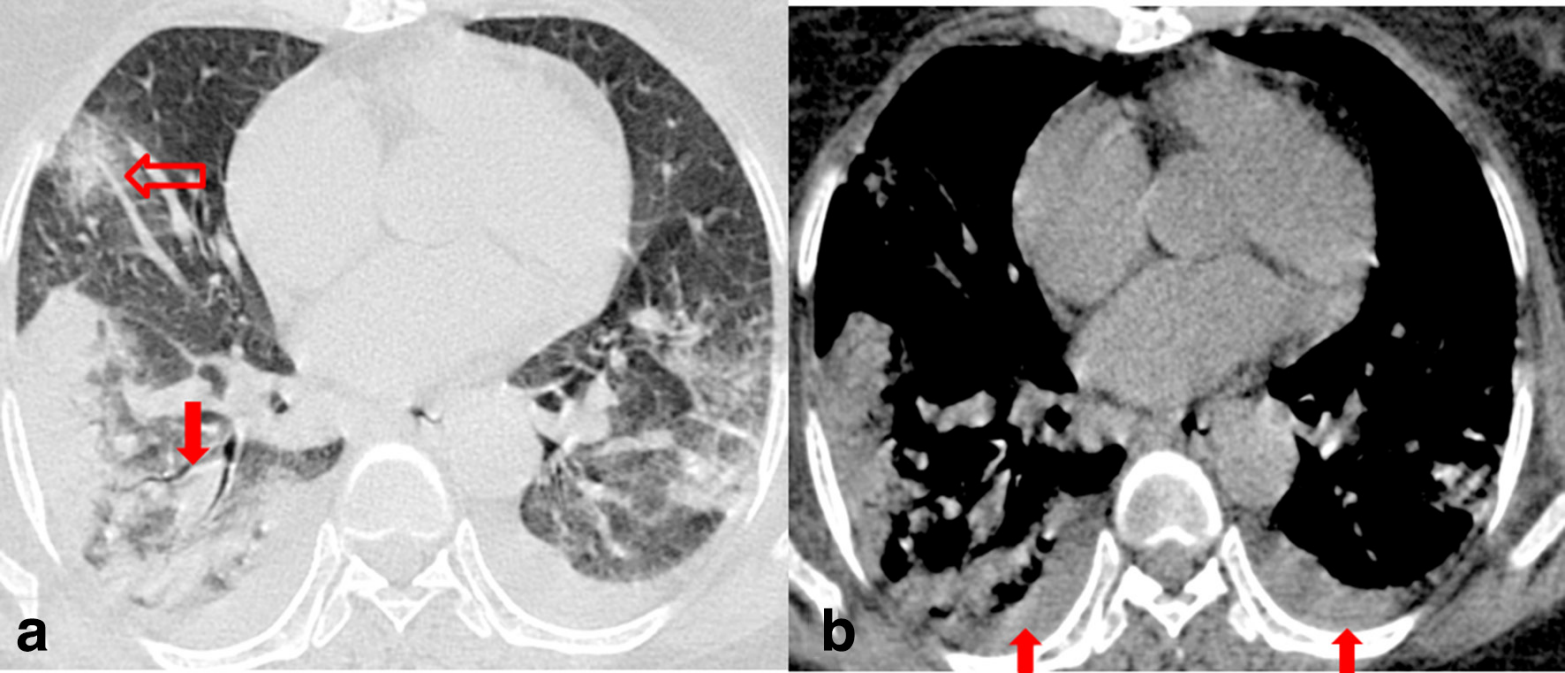
Non-contrast axial chest CT image (a) in lung window settings of a 60-year-old COVID-19 positive female patient, admitted in the ICU (clinically unstable), showing bilateral GGOs with consolidation on the right side. Air bronchogram sign is denoted by the solid arrow and vessel dilatation by a hollow arrow in (a). Axial CT image in the mediastinal window (b) shows bilateral mild pleural effusion denoted by arrows in (b). COVID-19, Corona virus disease 2019; GGO, ground glass opacity; ICU, intensive care unit.

With regards to the type of opacity, GGO was the dominant abnormality found in all 89 (100%) cases (Figure 4). The crazy paving pattern was seen in 70% (14/20) of unstable patients and in 21.7% (15/69) of clinically stable group with a statistically significant difference between the two groups (*p*-value < 0.05) (Figure 3). Consolidation patches were seen in 47.2% (42/89), significantly more frequent in the clinically unstable group when compared to the stable group (*p*-value < 0.05) (Figure 5). Paralleling the consolidative pattern, air bronchogram was seen in a high percentage of the clinically unstable group (Figure 6). Perilesional and/or intralesional segmental/subsegmental pulmonary vessel dilatation was seen in significantly higher proportion (90%) in clinically unstable patients requiring ICU admission or who subsequently expired (*p*-value < 0.05) (Figures 3 and 6).

**Figure 7.**
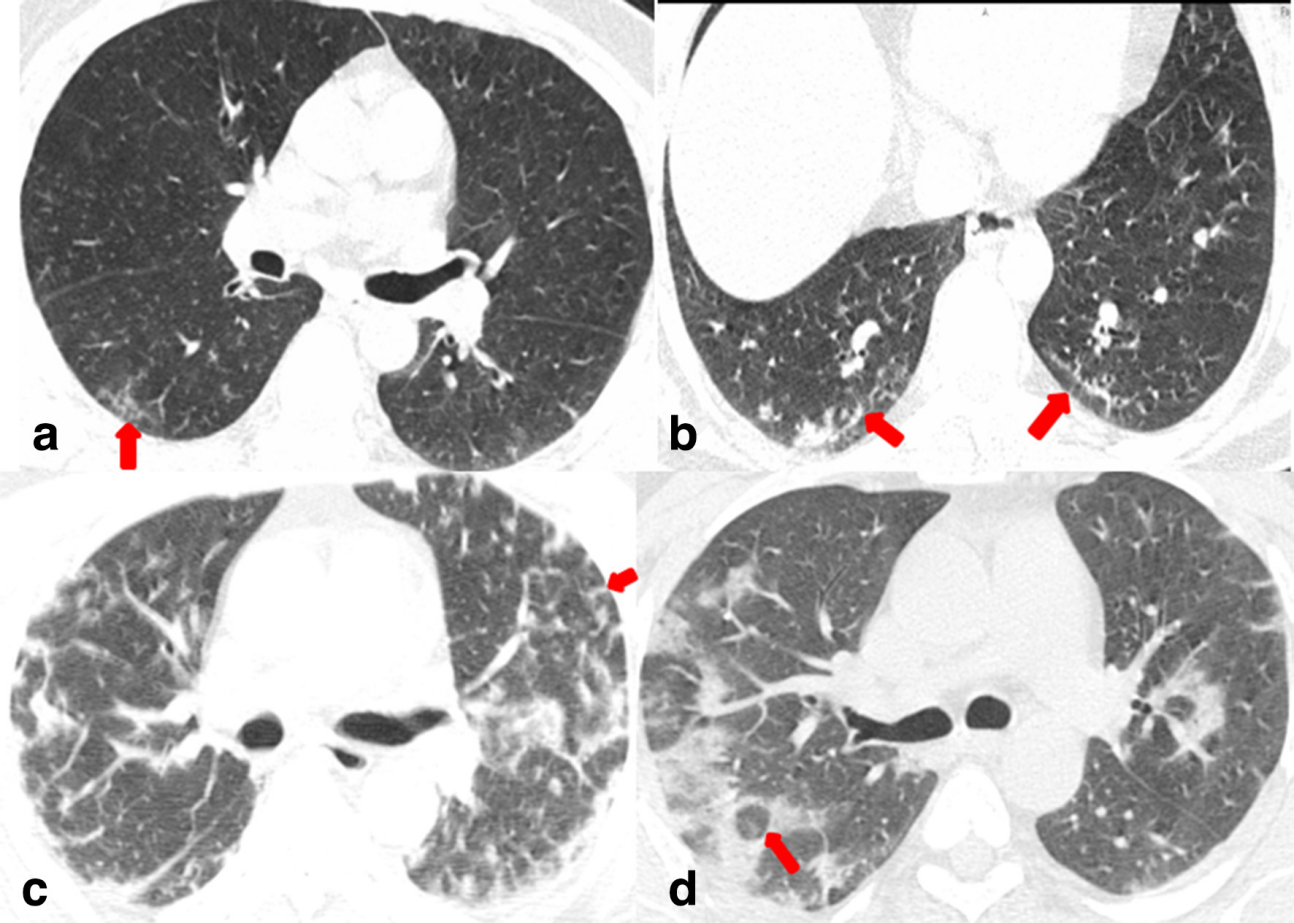
Non-contrast axial chest CT images in lung window settings in four different clinically stable (routine ward patients) COVID-19 positive patients: (a) 50-year-old male showing subpleural reticulations (arrow); (b) 56-year-old male showing subpleural curvilinear lines (arrows); (c) 61-year-old male showing multiple bilateral linear and reticular opacities in an arc pattern resembling peri-lobular opacities (arrow) typical of organizing pneumonia and (d) 49-year-old male showing bilateral multiple organizing consolidations with central lucency suggestive of reverse halo (atoll) sign (arrow).

Reticulations (26/89; 29.2%), atoll or reverse halo sign (16/89; 18%) and subpleural curvilinear lines (16/89; 18%) were seen slightly more frequently in the clinically stable group of patients than the unstable group. However, the difference between the two groups did not reach the level of statistical significance (Figure 7). Bronchial wall thickening, bronchodilatation, nodules, halo sign, pleural effusion and mediastinal lymphadenopathy were seen very infrequently. Pleural effusion was seen in three patient requiring ICU admissions and in none of the stable group (Figure 6). None of the patients showed pericardial effusion, centrilobular nodules or cavitation. Lung parenchymal abnormalities are summarized in Table 1.

## Discussion

Many patients with COVID-19 can be asymptomatic during early phase of the disease. However, a high viral load during the early phase renders these patients infectious to others.^2^ The paucity of information about the lurking enemy combined with the low sensitivity of RT-PCR prompted our CCC to undertake CT scanning in all consecutive patients, including those with minimal symptoms and those who were asymptomatic at admission. Additionally, reports about abnormal pulmonary findings in asymptomatic COVID-19 cases also partly influenced our decision to perform CT in asymptomatic cases. Inui et al^13^ reported 56% of asymptomatic COVID-19 cases with abnormal lung findings in Diamond Princess Cruise Ship. Bandirali et al^14^ reported pulmonary parenchymal abnormalities in 59% of asymptomatic or minimally symptomatic patients.

CT is a useful tool to detect COVID-19 pneumonia due to its rapidity, easy availability and swift interpretability. To the best of our knowledge, no imaging data of Indian patients is available, and the majority of results available have come mostly from China, Europe and USA. The majority (57.8%) of our COVID-19 positive cases had no lung parenchymal abnormalities at admission. Among these patients with negative CT, 36.9% were symptomatic and 63.1% were asymptomatic at admission. The high percentage (57.8%) of negative CT in our cohort is in stark contrast to studies from China, Korea and Italy, which reported lung parenchymal abnormalities in 61 to 100% COVID-19 positive patients.^15–21^ This discrepancy may have three plausible explanations. Firstly, it may be a consequence of a relatively young population in our study with a mean age of 47.7 years. Secondly, it may be reflective of less severity of the disease in our population which is also tentatively indicated by low mortality in our population so far. However, this result must be inferred with caution and should not falsely lead to complacency until it is substantiated by further studies. Thirdly, a low number of abnormal CT scans in our population may be because CT was performed in all consecutive RT-PCR positive patients regardless of symptoms as our healthcare was not overwhelmed.

The distribution and frequency of various lung abnormalities in our study cohort fairly corroborate the various recently published data.^15–21^ The majority of our COVID-19 cases showed predominance of GGOs with posterior and peripheral predilection with multilobar involvement. Bilaterality and lower lung zone involvement were frequently seen in our study. In a systematic review of imaging findings of 919 COVID-19 patients, Salehi et al^22^ reported that bilateral (87.5%) and multilobar (78.8%) lung involvement is common.

The primary goal of our study was to assess the findings on initial CT that could potentially predict features that adversely impact the patient outcome. Between two clinically distinct groups of stable patients (routine in-ward hospitalization) and unstable patients (ICU group/demised), age was adverse short-term prognostic factors as the mean age in ICU/demised group was significantly higher. Compared with symptoms of clinically stable (in-ward) patients, shortness of breath was more common (70% *vs* 4.3%) in clinically unstable patients at admission (Table 2).

With regards to the extent and distribution, presence of bilaterality and combined involvement of central–peripheral and AP lung involvement proved to be the harbingers of poor short-term clinical outcome as these were consistently seen in the clinically unstable group who required mechanical ventilation. A higher disease burden, assessed visually by its surrogate marker of percentage total lung involvement, also indicated a poor outcome in our study cohort.

Presence of crazy paving pattern, extensive consolidations and air bronchogram superimposed on the background of GGOs on initial chest CT examination increased the likelihood of patients deteriorating into clinically unstable group requiring ICU admission. These preliminary results indicate that increasing severity of pulmonary parenchymal damage in the form of increasing crazy paving pattern and coalescing consolidations with air bronchograms predisposes to clinically worse outcome. Pathologically, it may represent a continuum of increasing alveolar damage from minimal, represented by GGOs to diffuse, represented by extensive consolidations.

We observed segmental or subsegmental intralesional or perilesional pulmonary vessel enlargement in 90% of the clinically unstable patients which was significantly different from the clinically stable group. Our findings are in concurrence with Yan Li et al^16^ who reported vascular enlargement in 82.4%. Similarly, Caruso et al^21^ reported vessel enlargement in the majority of patients (89%). Small pulmonary vascular enlargement was reported to be frequently associated with COVID-19 pneumonia compared to non-COVID-19 pneumonia with a significant *p*-value (<0.001).^23^ So, the presence of vascular enlargement seems to be a specific additional feature of COVID-19 pneumonia. Vascular enlargement was speculated to occur due to the vasodilatory effect of proinflammatory cytokines or small pulmonary vessel embolism.^23^ Hemoptysis has been reported in COVID-19 pneumonia, with CT angiography subsequently demonstrating pulmonary embolism (PE) in these patients.^24^ Prospective Investigation of Pulmonary Embolism (PIOPED) group shows that only 13% of PE cases actually develop hemoptysis clinically.^24^ So, the true incidence of PE may be much higher in COVID-19 than is actually known at present.^25^ Alternatively, a distant possibility of infection induced secondary pulmonary vasculitis was proposed as a putative etiology for the vascular changes in COVID-19 pneumonia.^23^ Ackermann et al^26^ in an autopsy study, reported that COVID-19 pneumonia is associated with distinctive small pulmonary vascular changes comprising of severe endothelialitis associated with widespread microvascular thrombosis. The enlarged or thickened vessel sign observed in COVID-19 pneumonia on CT may be an imaging correlate of distinctive histopathological vascular changes reported in autopsy studies.

Though the exact pathophysiological mechanism of vascular enlargement is unclear, yet it conveys both diagnostic information and has prognostic implications. The enlarged vessel sign may help discriminate COVID-19 pneumonia from non-COVID-19 pneumonia which could be a vital piece of information and prognostically, vessel enlargement may predict a poor clinical outcome. Further autopsy studies focusing on pulmonary vascular changes in COVID-19 pneumonia may possibly answer these queries.

Recently, attempts have been made to study the radiological markers of prognosis in COVID-19 pneumonia. Li et al^15^ devised a semi-quantitative estimate of total lung involvement on CT which they referred to as total severity score (TSS) and concluded that TSS was significantly higher in severe-critical illness group compared to common illness group. Zhao et al^20^ found a statistical difference in age, presence of diffuse lung lesions, pleural effusion, architectural lung distortion and traction bronchiectasis between the non-emergency and emergency groups of COVID-19 patients. Yang et al^27^ used a quantitative method of the extent of lung opacification to compare radiological findings between severe and mild illness groups and concluded that CT severity scores were significantly higher in severe illness group. Colombi et al^12^ also devised a semi-quantitative method of estimation of the volume of the well-aerated lung (VOL-WAL) at baseline CT to determine the prognosis and observed that a combination of clinical parameters and %VOL-WAL are better prognostic indicators of COVID-19 pneumonia.

Reticulations, atoll or reverse halo sign and subpleural curvilinear lines were seen slightly more frequently in clinically stable group of patients than unstable group with no significant statistical difference. These changes presumably indicate an organizing pattern of pneumonia which needs further elucidation by long-term studies.^28^

There are a few limitations to our study. We focused on initial or baseline CT findings and did not perform follow-up CT examinations to look for temporal change (progression, stability or dissipation) in pulmonary abnormalities and also did not monitor the response to treatment. Secondly, we followed patients for a short period of time and did not study the long-term outcome of COVID-19 pneumonia. Third, important data like the presence or absence of underlying respiratory or cardiac comorbidities (or both), in the study population were not known. One could contend that some of the differences seen between the patients with severe and mild forms of disease could be partly due to underlying respiratory or cardiac comorbidities. For instance, GGO, interlobular septal thickening and pleural effusion could be due to underlying cardiac ailment or fluid overload rather than direct pulmonary damage caused by the virus. Further controlled studies may help differentiate the direct pulmonary effects from the infection from those caused by the underlying medical ailments. The small study population size is also a limitation. It is desirable to conduct large sample studies to validate our results and find any additional markers on initial imaging which could have the potential to predict short-term and long-term outcome in COVID-19 pneumonia.

## Conclusion

The present study corroborates the previously described CT features of COVID-19 pneumonia. A higher percentage of the total lung involvement, bilaterality, combined involvement of central–peripheral and AP lung (disease burden); crazy paving, consolidation with air bronchogram (alveolar damage) and segmental or subsegmental vessel enlargement (vascular changes) are present with increased frequency in the unstable patients requiring ICU admission and indicate a poor short-term prognosis. Segmental pulmonary vessel enlargement is a novel finding in COVID-19 pneumonia with unsubstantiated etiology.

## References

[1] World Health Organization Coronavirus disease 2019 (COVID-19): situation report. 119 2019.

[2] Chen Y, Li L SARS-CoV-2: virus dynamics and host response. Lancet Infect Dis 2020; 20: 515–6. doi: 10.1016/S1473-3099(20)30235-8 32213336 PMC7156233

[3] Huang C, Wang Y, Li X, Ren L, Zhao J, Hu Y, et al Clinical features of patients infected with 2019 novel coronavirus in Wuhan, China. Lancet 2020; 395: 497–506. doi: 10.1016/S0140-6736(20)30183-5 31986264 PMC7159299

[4] Kong W-H, Li Y, Peng M-W, Kong D-G, Yang X-B, Wang L, et al SARS-CoV-2 detection in patients with influenza-like illness. Nat Microbiol 2020; 5: 1–4. doi: 10.1038/s41564-020-0713-1 32265517

[5] Yang Y, Yang M, Shen C, et al Evaluating the accuracy of different respiratory specimens in the laboratory diagnosis and monitoring the viral shedding of 2019-nCoV infections. 2020;.

[6] Chung M, Bernheim A, Mei X, Zhang N, Huang M, Zeng X, et al Ct imaging features of 2019 novel coronavirus (2019-nCoV. Radiology 2020; 295: 202–7. doi: 10.1148/radiol.2020200230 32017661 PMC7194022

[7] Bernheim A, Mei X, Huang M, Yang Y, Fayad ZA, Zhang N, et al Chest CT findings in coronavirus Disease-19 (COVID-19): relationship to duration of infection. Radiology 2020; 295: 200463. doi: 10.1148/radiol.2020200463 32077789 PMC7233369

[8] Ye Z, Zhang Y, Wang Y, Huang Z, Song B, et al Chest CT manifestations of new coronavirus disease 2019 (COVID-19): a pictorial review. Eur Radiol 2020; 382. doi: 10.1007/s00330-020-06801-0 PMC7088323 32193638

[9] Tabatabaei SM, Talari H, Moghaddas F, Rajebi H Computed tomographic features and short-term prognosis of coronavirus disease 2019 (COVID-19) pneumonia: a single-center study from Kashan, Iran. Radiology: Cardiothoracic Imaging; 2020 Apr 20;2(2):e200130. 10.1148/ryct.2020200130 PMC7233449 33778569

[10] World Health Organization Clinical management of severe acute respiratory infection when novel coronavirus (nCoV) infection is suspected: interim guidance.. , 2020 Updated on March 13, 2020Published on January 12 Available from: https://www.who.int/publications-detail/clinical-management-of-severe-acute respiratory-infection-when-novel-coronavirus-(ncov)-infection-is-suspected [Accessed on March 20, 2020].

[11] Hansell DM, Bankier AA, MacMahon H, McLoud TC, Müller NL, Remy J Fleischner Society: glossary of terms for thoracic imaging. Radiology 2008; 246: 697–722. doi: 10.1148/radiol.2462070712 18195376

[12] Colombi D, Bodini FC, Petrini M, Maffi G, Morelli N, Milanese G, et al Well-aerated lung on admitting chest CT to predict adverse outcome in COVID-19 pneumonia. Radiology 2020; :: 201433 Apr 17. doi: 10.1148/radiol.2020201433 PMC7233411 32301647

[13] Inui S, Fujikawa A, Jitsu M, Kunishima N, Watanabe S, Suzuki Y et al Chest CT findings in cases from the cruise ship “Diamond Princess” with coronavirus disease 2019 (COVID-19. Radiology: Cardiothoracic Imaging; 2020 Mar 17;2(2):e200110. 10.1148/ryct.2020204002 PMC7233437 33779623

[14] Bandirali M, Sconfienza LM, Serra R, Brembilla R, Albano D, Pregliasco FE, et al Chest radiograph findings in asymptomatic and minimally symptomatic quarantined patients in Codogno, Italy during COVID-19 pandemic. Radiology 2020; 295: E7. doi: 10.1148/radiol.2020201102 32216718 PMC7233394

[15] Li K, Fang Y, Li W, Pan C, Qin P, Zhong Y, et al Ct image visual quantitative evaluation and clinical classification of coronavirus disease (COVID-19. Eur Radiol 2020; 54 Mar 25:1-0. doi: 10.1007/s00330-020-06817-6 PMC7095246 32215691

[16] Li Y, Xia L Coronavirus disease 2019 (COVID-19): role of chest CT in diagnosis and management. AJR Am J Roentgenol 2020; 214 :: 1–7 Feb 21. doi: 10.2214/AJR.20.22954 32130038

[17] Huang G, Gong T, Wang G, Wang J, Guo X, Cai E, et al Timely diagnosis and treatment shortens the time to resolution of coronavirus disease (COVID-19) pneumonia and lowers the highest and last CT scores from sequential chest CT. AJR Am J Roentgenol 2020; 30: 1–7. doi: 10.2214/AJR.20.23078 32223665

[18] Zhu Y, Gao Z-H, Liu Y-L, Xu D-Y, Guan T-M, Li Z-P, DY X, ZP L, et al Clinical and CT imaging features of 2019 novel coronavirus disease (COVID-19. J Infect 2020; 81: 147–78. doi: 10.1016/j.jinf.2020.03.033 PMC7194958 32277968

[19] Huang C, Wang Y, Li X, Ren L, Zhao J, Hu Y, et al Clinical features of patients infected with 2019 novel coronavirus in Wuhan, China. The Lancet 2020; 395: 497–506. doi: 10.1016/S0140-6736(20)30183-5 PMC7159299 31986264

[20] Zhao W, Zhong Z, Xie X, Yu Q, Liu J Relation between chest CT findings and clinical conditions of coronavirus disease (COVID-19) pneumonia: a multicenter study. AJR Am J Roentgenol 2020; 214: 1072–7. doi: 10.2214/AJR.20.22976 32125873

[21] Caruso D, Zerunian M, Polici M, Pucciarelli F, Polidori T, Rucci C, et al Chest CT features of COVID-19 in Rome, Italy. Radiology 2020;: 201237. doi: 10.1148/radiol.2020201237 PMC7194020 32243238

[22] Salehi S, Abedi A, Balakrishnan S, Gholamrezanezhad A, disease C COVID-19): a systematic review of imaging findings in 919 patients. American Journal of Roentgenology 2019;. 14: 1–7 2020 Mar. 10.2214/AJR.20.23034 32174129

[23] Parry AH, Wani AH Segmental pulmonary vascular changes in COVID-19 pneumonia. American Journal of Roentgenology 2020; 8: W1. doi: 10.2214/AJR.20.23443 32383969

[24] Casey K, Iteen A, Nicolini R, Auten J COVID-19 pneumonia with hemoptysis: acute segmental pulmonary emboli associated with novel coronavirus infection. Am J Emerg Med 2020;08 Apr 2020. doi: 10.1016/j.ajem.2020.04.011 PMC7141630 32312574

[25] Parry AH, Wani AH Pulmonary embolism in coronavirus disease-19 (COVID-19) and use of compression ultrasonography in its optimal management. Thromb Res 2020; 192: 36. doi: 10.1016/j.thromres.2020.05.022 32425262 PMC7229911

[26] Ackermann M, Verleden SE, Kuehnel M, Haverich A, Welte T, Laenger F, et al Thrombosis, and angiogenesis in Covid-19. New England Journal of Medicine 2020;. 10.1056/NEJMoa2015432 PMC7412750 32437596

[27] Yang R, Li X, Liu H, Zhen Y, Zhang X, Xiong Q et al Chest ct severity score: An imaging tool for assessing severe covid-19. Radiology: Cardiothoracic Imaging; 2020 Mar 30;2(2): e200047. 10.1148/ryct.2020200047 PMC7233443 33778560

[28] Wang Y, Dong C, Hu Y, Li C, Ren Q, Zhang X, et al Temporal changes of CT findings in 90 patients with COVID-19 pneumonia: a longitudinal study. Radiology 2020;: 200843. doi: 10.1148/radiol.2020200843 PMC7233482 32191587

